# Differential Bees Flux Balance Analysis with OptKnock for *In Silico* Microbial Strains Optimization

**DOI:** 10.1371/journal.pone.0102744

**Published:** 2014-07-21

**Authors:** Yee Wen Choon, Mohd Saberi Mohamad, Safaai Deris, Rosli Md. Illias, Chuii Khim Chong, Lian En Chai, Sigeru Omatu, Juan Manuel Corchado

**Affiliations:** 1 Artificial Intelligence and Bioinformatics Group, Faculty of Computing, Universiti Teknologi Malaysia, Johor, Malaysia; 2 Department of Bioprocess Engineering, Faculty of Chemical Engineering, Universiti Teknologi Malaysia, Skudai, Johor, Malaysia; 3 Department of Electronics, Information and Communication Engineering, Osaka Institute of Technology, Osaka, Japan; 4 Biomedical Research Institute of Salamanca/BISITE Research Group, University of Salamanca, Salamanca, Spain; University of Georgia, United States of America

## Abstract

Microbial strains optimization for the overproduction of desired phenotype has been a popular topic in recent years. The strains can be optimized through several techniques in the field of genetic engineering. Gene knockout is a genetic engineering technique that can engineer the metabolism of microbial cells with the objective to obtain desirable phenotypes. However, the complexities of the metabolic networks have made the process to identify the effects of genetic modification on the desirable phenotypes challenging. Furthermore, a vast number of reactions in cellular metabolism often lead to the combinatorial problem in obtaining optimal gene deletion strategy. Basically, the size of a genome-scale metabolic model is usually large. As the size of the problem increases, the computation time increases exponentially. In this paper, we propose Differential Bees Flux Balance Analysis (DBFBA) with OptKnock to identify optimal gene knockout strategies for maximizing the production yield of desired phenotypes while sustaining the growth rate. This proposed method functions by improving the performance of a hybrid of Bees Algorithm and Flux Balance Analysis (BAFBA) by hybridizing Differential Evolution (DE) algorithm into neighborhood searching strategy of BAFBA. In addition, DBFBA is integrated with OptKnock to validate the results for improving the reliability the work. Through several experiments conducted on *Escherichia coli*, *Bacillus subtilis*, and *Clostridium thermocellum* as the model organisms, DBFBA has shown a better performance in terms of computational time, stability, growth rate, and production yield of desired phenotypes compared to the methods used in previous works.

## Introduction

In the recent years, more accurate genome annotation and more complete pathway information, promising achievements have been made on *in silico* metabolic network reconstruction [1]. In addition, one of the most comprehensive stoichiometric models built upon the genome of *E. coli* had been available [2]. Currently, the new emerging study is aiming to computationally optimize the microbial strains for the overproduction of particular chemical and biochemical compounds, diversely ranging from industrial interests to environmental usages [3]. Computational models are of the central importance for the investigation of general biological functions and applications in the area of biomedicine and biotechnology, this accelerates the process of developing computational models to simulate the actual processes inside the cells [4]. Retrofitting of the cellular metabolism is essential, as naturally, due to the cellular metabolism responses in the history of selective pressures, microorganisms evolve by optimizing their growth rather than having overproduction of specific chemical compounds. Classical strain improvement such as random mutagenesis and screening are among traditional methods to retrofit the microbial metabolism. However, the effects of genetic modification on the desirable phenotypes are difficult to predict due to the complexities of the metabolic networks that lead to data ambiguity. Furthermore, the combinatorial problem that is caused by a large number of reactions in cellular metabolism makes the process to obtain optimal gene deletion strategies challenging. Basically, a genome-scale metabolic model is usually large in size. Another problem in the current metabolic engineering field is as the size of the problem increases, the computation time increases exponentially. In later years, metabolic engineering is introduced to retrofit microbial metabolism. In metabolic engineering, the main objective is to increase the production of particular chemical and biochemical compounds through genetic engineering. Gene knockout is one of the most common genetic engineering techniques for the overproduction of particular chemical and biochemical compounds, in which one of the organism’s genes is made inoperative. Currently, this technology has been successfully applied in many organisms, from unicellular eukaryotes to mammals, including human cells.

In the recent years, the study on computational algorithms to identify optimal gene knockout strategies for obtaining improved phenotypes is growing rapidly. The first rational modeling framework (known as OptKnock) is introduced by Burgard *et al.*, for predicting gene knockout strategies aiming to the overproduction of a desired metabolite. Without affecting the operation of the internal flux distribution, OptKnock identifies a set of gene (reaction) deletions to maximize the flux of a desired metabolite so that the growth or another objective function is optimized [5].

OptKnock uses mixed integer linear programming (MILP) to formulate a bi-level linear optimization that is promising to find the global optimal solution. OptGene is extended from OptKnock which formulates the *in silico* design problem by using Genetic Algorithm (GA) [6]. Meta-heuristic methods are usually known to be able to produce near-optimal solutions with reasonable computation time, which is favourable, and the objective function that can be optimized is flexible. OptGene is available in two representation schemes which are binary and integer. The binary representation is more complex and leads to a solution with a large number of knockouts even though it is closer to the natural evolution of microbial genomes. The integer representation gives a more compact genome but the problem is that a priori number of gene knockouts need to be defined [7]. To overcome the problems, Rocha et al. [7] proposed two optimization algorithms: Simulated Annealing (SA) and Set-based Evolutionary Algorithms (SEAs) to allow the automatic finding of the best number of gene deletions to achieve a given productivity goal. The performance to identify optimal gene knockout strategies still needs improvement.

A hybrid of BA and FBA (BAFBA) was proposed by Choon *et al.* [8], which showed a better performance in predicting optimal gene knockout strategies in terms of growth rate and production yield. Bees Algorithm (BA) was first introduced by Pham *et al.* [9], which is a typical meta-heuristic optimization approach that has been applied to several problems, such as controller formation [10], image analysis [11], and job multi-objective optimization [12]. BA mimicks the intelligent behaviors of honeybees. It locates the most promising solutions, and selectively explores its neighborhoods for the global maximum of the objective function. According to a series of recent publications, BA is efficient in solving optimization problems [9]–[12]. Nevertheless, BA is relatively weak in local search activities due to its dependency on random search [13]. In this paper, DBFBA, a hybrid of DE algorithm into the neighborhood searching strategy of BAFBA has been proposed to improve the performance of BAFBA. DE algorithm is a heuristic approach mainly having three advantages; finding the true global minimum regardless of the initial parameter values, fast convergence, and using few control parameters. In addition, to improve the reliability of the work, the OptKnock was integrated into DBFBA to validate the results rather than validating the results only through literature. OptKnock is a well-recognized modelling framework, which is widely used in assisting biologists for gene knockout experiments in laboratory. It is proven that predicted strains from OptKnock can lead to successful production strains and that adaptive evolution of the engineered strains can lead to improved production capabilities [14]. Besides, the previous work, OptGene, integrated with OptKnock to validate the results too, hence OptKnock is used to validate the results in this paper [7]. This paper shows that DBFBA is capable of solving large size problems in short computational time as well as improves the performance in predicting optimal gene knockout strategies. The results obtained by DBFBA in four case studies are also presented where *E.coli* (*Escherichia Coli*) *i*JR904 model, *B.subtilis* (*Bacillus subtilis*) model, and *C. thermocellum* (*Clostridium thermocellum*) model are the target microorganisms [2], [15]–[16]. In addition, a computational benchmarking analysis was also conducted to test the performance of the hybrid of Bee algorithm and DE algorithm.

This paper is organized as follows: Firstly, problem formulation is introduced, and the details of the BAFBA and the proposed DBFBA are described. The validation analyses by using OptKnock are also explained. Then, the datasets and experimental setup are described. Next, experimental results are presented. Then, the discussion on the obtained results is addressed, which deliberates the contributions of this work. Lastly, this paper is summarized by providing main conclusion and future developments.

## Materials and Methods

### Problem Formulation

The problem to identify optimal gene knockout strategies from the biological models can be formulated as follows: Suppose a model which contains the stoichiometric matrix **S** provides the linear relationship of the model between the flux rates of the reactions (**v**) and the derivatives of the reactant concentrations (**x**). The matrix is a constant, while the flux vector is a variable. Assume that there are *m* reactants and *n* reactions between them.

Flux vector:
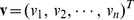
(1)


Concentration vector:
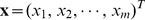
(2)


Dynamic mass balance equation:
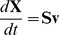
(3)where ***T*** represents the time.

The chemical elements, ionic charge, and biochemical moieties must be balanced in the stoichiometric matrix. The objective is to find the optimal gene knockout strategy which can improve the product yields of industrially important chemicals, while sustaining the growth rate of the microorganism. This is commonly performed by using the linear programming, defined as follows:
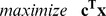


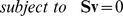


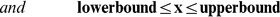
(4)where **v** represents the vector of fluxes, **S** is the stoichiometric matrix. The expression (**c^T^x**) to be maximized or minimized is known as the objective function, where **c** is a vector of weights, indicating contributions of each reaction to the objective function. The inequalities of lower bound and upper bound define the maximal rates of flux for every reaction corresponding to the columns of the stoichiometric matrix.

### A hybrid of BA and FBA (BAFBA)

**Figure 1:**
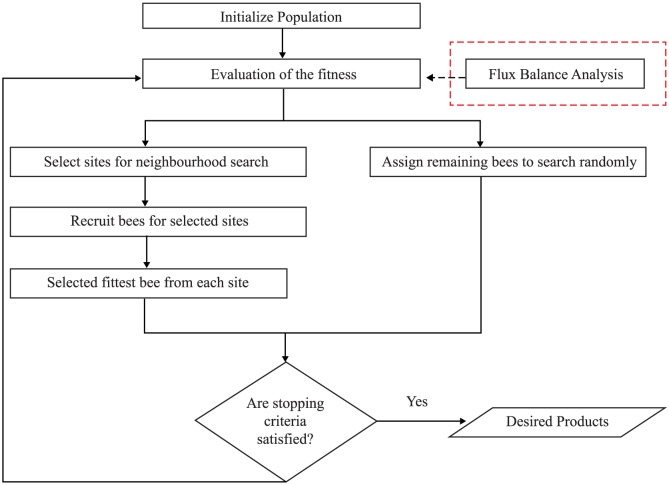
BAFBA Flowchart. Note: Red-dotted box is Flux Balance Analysis which is hybridized into standard BA as an objective function in order to predict the effect of gene knockout.

Figure 1 shows the flow of BAFBA. BAFBA is initialized by mimicking a population of bees. In identifying gene knockout strategies, a bee is represented by a binary variable to indicate the absence or the presence of genes in the reaction. In this study, the BAFBA is started with the bees being placed randomly in the search space. The fitness of the sites visited by the bees is evaluated using FBA. Bees with the highest fitness would be denoted as ‘selected bees’ and the sites visited by them would be chosen for neighbourhood search. Small amount of ‘selected bees’ was expected to encourage local exploitation. After performing many tests, it was found that appropriate maximum ‘selected bees’ was (1/4)*×n*. The amount of selected bees was limited within the range [1,(1/4)*×n*] to prevent unnecessary selection of sites for neighborhood search. Each bee was required to go through this repetitive local search neighborhood procedure until the best possible answer obtained. Meanwhile, the remaining bees were assigned randomly to search for new potential solutions.

Before attempting to propose DBFBA, it is crucial to find the limitations of BAFBA [9]. BA dependence on random search makes it relatively weak in local search activities and it suffers of slow convergence due to the repetitive iteration of the algorithm. Repetition of unnecessary similar process in neighborhood search causes an additional computational time in generating solution.

### Differential Bees Flux Balance Analysis (DBFBA) with OptKnock

**Figure 2:**
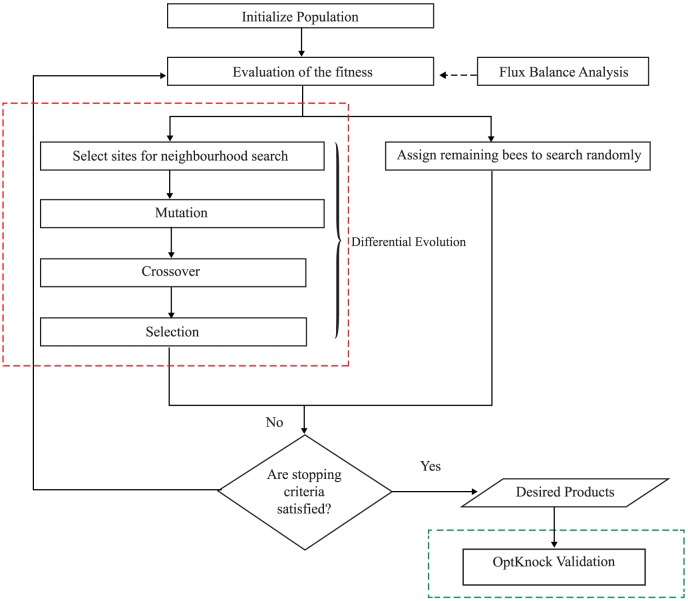
Overall framework of DBFBA with OptKnock. Note: Red-dotted box is DE algorithm that is hybridized into BAFBA in order to improve the local search performance of BAFBA. Green-dotted box is OptKnock validation that has been integrated into DBFBA in this paper.

**Figure 3:**
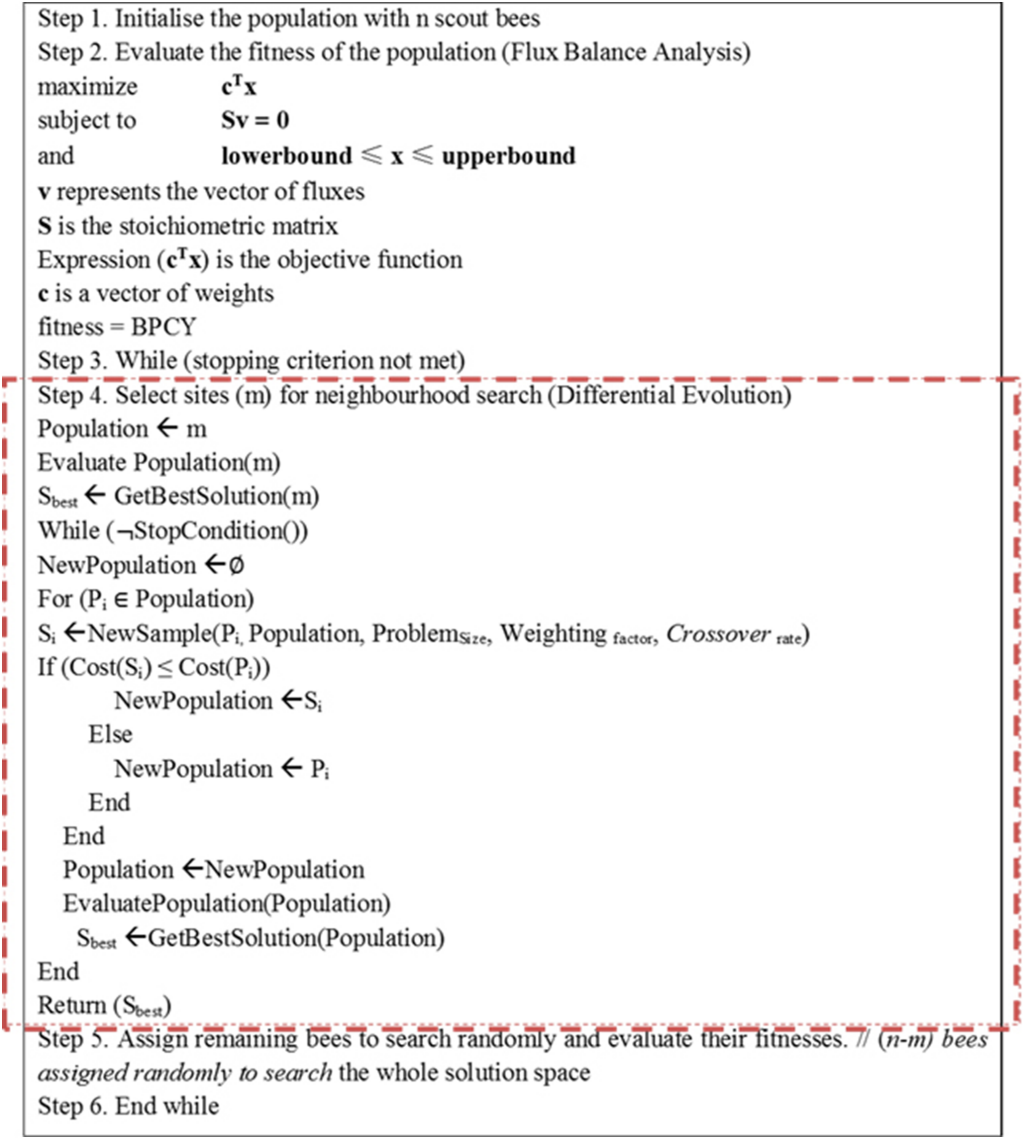
Pseudo code of DBFBA.

In this paper, DBFBA with OptKnock for identifying optimal gene knockout strategy was proposed to overcome the limitations of BAFBA and previous works [5]–[7], [9]. DBFBA in this work differs from the BAFBA in the neighborhood search activities. The proposed DBFBA improved the operation by hybridizing DE algorithm into BAFBA. In addition, OptKnock was integrated into DBFBA to improve the reliability of the results. Figure 2 shows the overall framework of DBFBA and Figure 3 is the pseudo code of DBFBA. The important steps are explained in the following subsections.

#### Bee Representation of Metabolic Genotype

**Figure 4:**
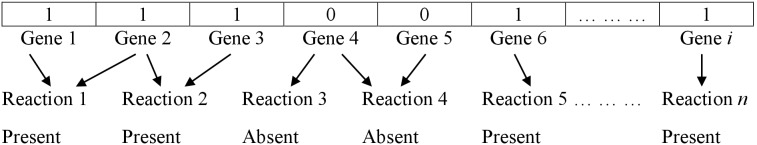
Bee representation of metabolic genotype [6].

One or more genes can be discovered in each reaction in a metabolic model. In this paper, each of those genes is represented by a binary variable, where 0 represents the absence of the gene and 1 represents the presence of the gene in the reaction. These variables form a ‘bee’ representing a specific mutant that lacks some metabolic reactions when compared with the wild type (Figure 4).

#### Initialization of the Population

The algorithm starts with an initial population of *n* scout bees. Each bee is initialized as by assuming a reaction with *n* genes. Bees in the population are initialized by setting present or absent status to each gene randomly. Initialization of the population is done randomly so that all bees in the population have an equal chance to be selected. Specific rules like co-regulated genes tending to be clustered did not affect the result of selecting the genes. The result might not truly reflect the population if it is done with bias setting.

#### Flux Balance Analysis

Each site is given a fitness score that determines whether to recruit more bees or should be abandoned. In this work, FBA has been used to calculate the fitness score for each site (refer to Equation (4)). In the study made by García Sánchez *et al.* [17], the best predictions were obtained using “maximization of growth”, and with some combinations that included this objective. Hence, in this paper, maximization of growth is applied. After maximizing the cellular growth, mutant with a growth rate of more than 0.1 continues the process by maximizing the desired product flux at fixed optimal cellular growth value. After conducting a small number of trial, the optimal cellular growth value was fixed at 90% from the value obtained from FBA, since the production yield of the desired metabolite is always 0 when the growth is at maximum. Production yield is the maximum amount of product that can be generated per unit of substrate. The following shows the calculation for production yield:
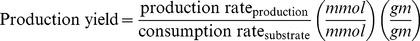
(5)where mmol = millimole and gm is gram.

We used Biomass-product coupled yield (BPCY) as the fitness score in this work. According to Soons *et al.* (2013), metabolic networks can function in living cells under various biological objectives, depending on the relevant organism and its genetic and environmental context. However, biological objectives have been clarified as only applicable for analyzing a number of organisms in terms of the microbial metabolic engineering. It is desirable to couple the formation of the desired product to growth [18]. The calculation for BPCY is as follows:
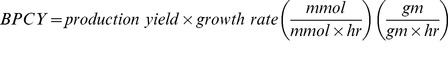
(6)Where mmol is millimole, hr is hour and gm is gram.

**Figure 5:**
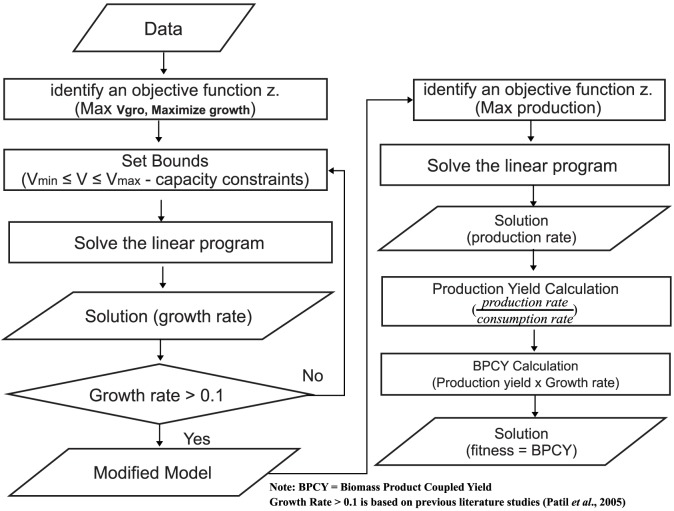
The flow of fitness calculation.

The flow of the fitness calculation is showed in Figure 5.

#### Neighborhood Search (Differential Evolution Algorithm)

This algorithm carries out neighbourhood searches in the favored sites (*m*) by using DE algorithm. DE algorithm operates by maintaining a population of candidate solutions and creating new candidate solutions through the mutation and crossover operation of DE, and keeps the fitness candidate solution. In this paper, the candidate solutions are the *m* favored sites from the population initialized by using BA. The algorithm starts with the solution, then goes through the mutation and crossover operation to create new candidate solutions. Since the candidate solutions are initialized by BA randomly, hence the specific rules on the distribution of gene on chromosome did not affect the result for neighbourhood gene selection too. The mutation factor F is a constant from [0,2] and elements of the candidate solutions enter the new candidate solutions with probability *CR*. In this paper, *m* is equal to 15, F is equal to 1, and *CR* is equal to 0.5. The values are obtained by conducting a small number of trials with the range of 10 to 25, 0 to 2, and 0.1–0.9, respectively. This step is important as there might be better solutions than the original solution in the neighborhood area.

#### Random Assignment and Termination

The remaining bees in the population are sent randomly around the search space to scout for new feasible solutions. This step is done randomly to avoid overlooking the potential results that are not in the range. These steps are repeated until either the maximum loop value is met or the fitness function has converged. In the end, the colony generates two parts to its new population – representatives from each selected patch and other scout bees assigned to perform random searches. There is no necessities to shrink the final result from DBFBA, there will be no additional genes to the solutions, because the fitness calculation is based on the bees that consist of a set of genes and it is not calculate the effect of each gene, which may contribute to the production of desired phenotypes.

#### OptKnock Validation

**Figure 6:**
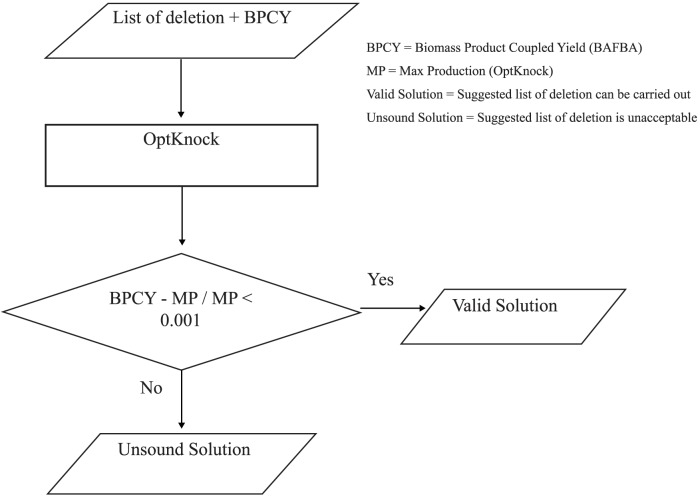
The flow of OptKnock validation.

In this paper, OptKnock [5] has been used to evaluate the result obtained through the list of gene knockout from DBFBA. If the difference between the BPCY obtained from DBFBA and the maximum production rate obtained by OptKnock are less than 0.001, the list would be considered as a valid solution. Figure 6 shows the flow of the validation.

## Datasets and Experimental Setup

There are three models used to test the operation of DBFBA in this work, which are *E. coli*, *B. subtilis*, and *C. thermocellum* models, respectively. The three models are well-established models, the models are developed and used to study the bacterium’s metabolism and phenotypic behaviour. Therefore the distribution of the genes (clustered/location) has no impact in our works since our works are not dealing with the expression patterns of the genes. All the models are pre-processed through several steps based on biology assumptions and computational approaches before it was applied to DBFBA. Lethal reactions such as genes that are found to be lethal *in vivo*, but not *in silico*, should be removed to improve the quality of the results. The results are invalid if a lethal reaction is deleted. In addition, dead-end reactions that cannot be activated within a feasible flux distribution when considering the entire universal reactions set are removed. The first model is *E. coli* model that contains 904 genes, 931 unique biochemical reactions, and 761 metabolites [2] (http://bigg.ucsd.edu/). *E. coli i*JR904 has been used in this work to test the reliability of DBFBA as this model was also used in previous works [5], [7]. After the pre-processing step, the size of the model is reduced to 667 reactions. The second model is *B. subtilis i*Bsu1103 model [15] (http://genomebiology.com/content/supplementary/gb-2009-10-6-r69-s4.xml) that includes 1437 reactions associated with 1103 genes. This model has been preprocessed and the size is reduced to 763 reactions. The last model is *C.thermocellum* (ATCC 27405) *i*SR432 model [16] (http://www.biomedcentral.com/content/supplementary/1752-0509-4-31-s3.xml) that contains 577 reactions representing the function of 432 genes. This model underwent the pre-processing step and the size has reduced to 351 reactions. This work has generated three results, which are the list of knockout genes, growth rate, and BPCY (Biomass Product Coupled Yield). Unit for growth rate is hour^−1^ while unit for BPCY is in milli-gram (gram-glucose.hour)^−1^. The experiments were conducted using a 2.3 GHz Intel Core i7 processor with 8 GB RAM workstation.

The results were compared with the previous works as reported in the literature studies [5], [7], [9]. The experiment carries out 100 individual runs to test on DBFBA with OptKnock, in which the result shown is the best result among the runs.

## Results and Discussion

### Benchmark Functions

In this paper, an improved method, DBFBA, was proposed to test the performance of DBFBA. For evaluation, a benchmarking analysis was conducted. However, benchmark functions could only be tested on DB and BA, as FBA is an objective function. Hence, the benchmark functions on DB and BA in this study were tested. As BA is used to attain the maximum, the functions are inverted before the algorithm is applied. The De Jong, Martin & Gaddy, Branin, and Griewangk functions have been used in this study. A total of 100 individual runs have been carried out to test on DB and BA.

Table 1 shows the mathematical representation of the functions. Table 2 shows the mean and standard deviation (STD) of all the functions, De Jong, Martin & Gaddy, Branin, and Griewangk, tested on both original BA and DB. The results showed that DB performs better than BA. All functions obtained low STD, indicating that the result from each run is very close to the mean. As a conclusion, the stability of the proposed method is high as the difference between the result for each individual run is small. In addition, the means for both algorithms are similar, indicating that DB is indeed reliable, as the results obtained from DB are consistent with the results from previous works.

**Table 1:** Mathematical representation of De Jong Martin & Gaddy, Schwefel, and Griewangk functions.

Name	Mathematical representation
De Jong	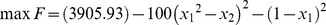
Martin & Gaddy	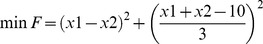
Branin	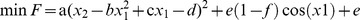 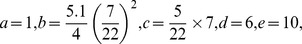 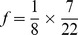
Griewangk	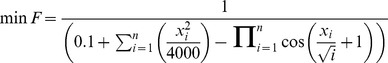

**Table 2:** Obtained fitness value of all benchmark functions.

Function	Mean		STD	
	BA	DB	BA	DB
De Jong	3.91e+03	3.91E+03	0.000504	**2.13793E-05**
Martin & Gaddy	11.1083	11.1111	0.002797	**0**
Branin	26.5619	26.5537	0.02917	**0.02771**
Griewangk	–0.5263	–0.5263	5.76765E-09	**0**

Note: The bold numbers represent the best result.

### Production of Succinic Acid and Lactic Acid by *E.coli*

Tables 3 and 4 summarize the results obtained from DBFBA for succinic acid and lactic acid production in *E.coli*. As shown from the results, this method produces better results than the previous works in terms of growth rate and BPCY, and can also identify potential genes that can be removed [5], [7], [9].

**Table 3:** Comparison between different methods for growth rate and BPCY Succinic acid by *E.coli.*

Method	GrowthRate (1/hr)	BPCY	List of knockout genes
DBFBA	**0.74529**	**13.58**	ENO, PFL, PYK**
BAFBA [9]	0.58512	3.84893	FUM**, PTAr**, RPE
SA + FBA [7]	N/A	0.35785	MALS, ORNDC, FUM**, GLYCL, GHMT2, ADPT, DCYTD, DUTPDP, URIDK2r, NTD8, PUNPI, THD2, GND, PFL, SUCFUMt
OptKnock [5]	0.31	N/A	PYK, ACKr, PTAr**, Phosphotransferase system

Note: The bold numbers represent the best result. N/A – Not Applicable.

*Common genes for all methods.

**Common genes in either 2 methods. BPCY is in gram (gram-glucose.hour)^−1^.

**Table 4:** Comparison between different methods for growth rate and BPCY of Lactic acid by *E.coli.*

Method	Growth Rate (1/hr)	BPCY	List of knockout genes
DBFBA	**0.86719**	**16.1905**	ACALD*, MDH, TALA
BAFBA [9]	0.58586	3.5656	GAPD, L_LACD2, PTAr**
SA + FBA [7]	N/A	0.39850	ACLD19**, DRPA, GLYCDx, F6PA, TPI, LDH_D2, EDA, TKT2, LDH_D-
OptKnock [5]	0.28	N/A	ACKr, PTAr**, ACALD**

Note: The bold numbers represent the best result. N/A – Not Applicable.

*Common genes for all methods.

**Common genes in either 2 methods. BPCY is in gram (gram-glucose.hour)^−1^.

**Figure 7:**
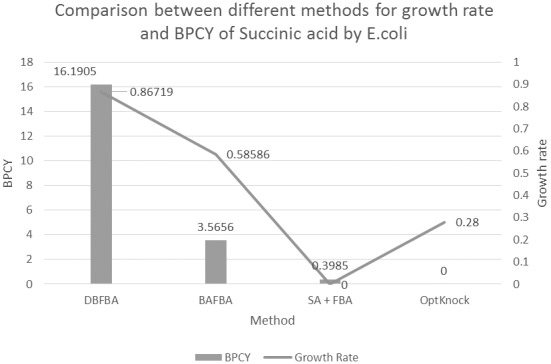
Comparison between different methods for growth rate and BPCY of Succinic acid by *E.coli*. Note: BPCY is in gram (gram-glucose.hour)^−1^. Unit for growth rate is hr^−1^.

**Figure 8:**
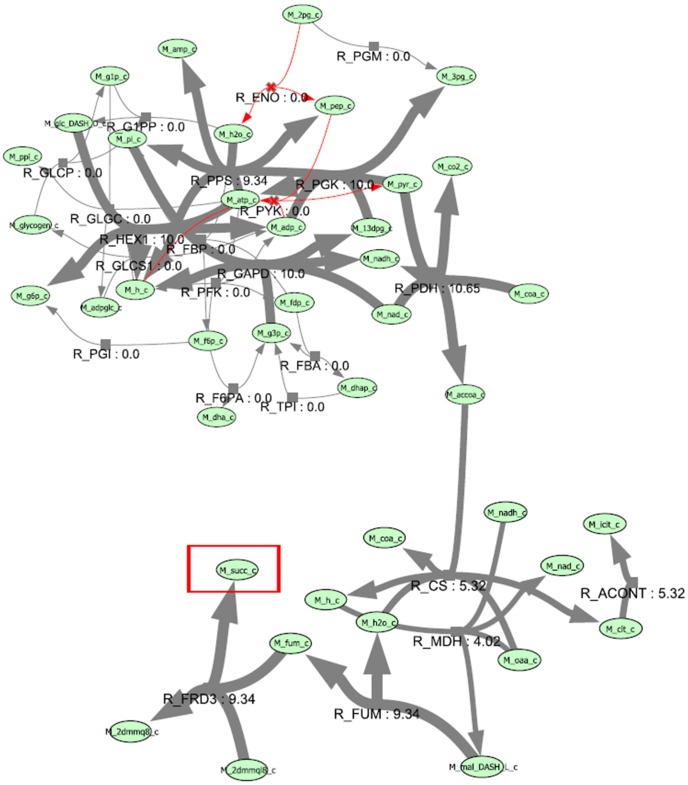
Flux map of the production of succinic acid.

Table 3 shows DBFBA for the removal of three reactions from the network results in succinic acid growth rate reaching 0.85512 and BPCY reaching 14.2907, which is better than previous works. The removal of enolase (ENO) affects the phosphotransferase system, which causes the network to rely exclusively on glucokinase for the uptake of glucose. In addition, the removal of pyruvate kinase (PYK), makes PEP carboxykinase as the only central metabolic reaction capable of draining a significant amount of PEP supplied by glycolysis [5]. Figure 7 presents the comparison between different methods for growth rate and BPCY of succinic acid. Figure 8 shows the flux map for the production of succinic acid. The flux map shows that there is an increase in the flux to succinic acid.

**Figure 9:**
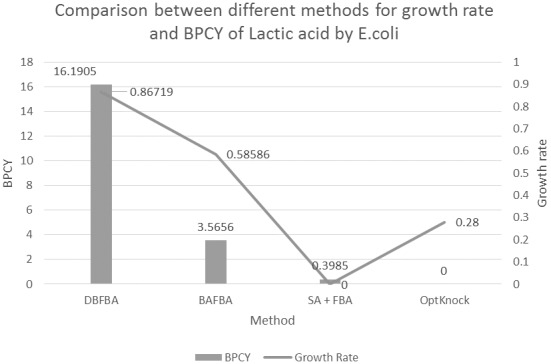
Comparison between different methods for growth rate and BPCY of Lactic acid by *E.coli.* Note: BPCY is in gram (gram-glucose.hour)^−1^. Unit for growth rate is hr^−1^.

**Figure 10:**
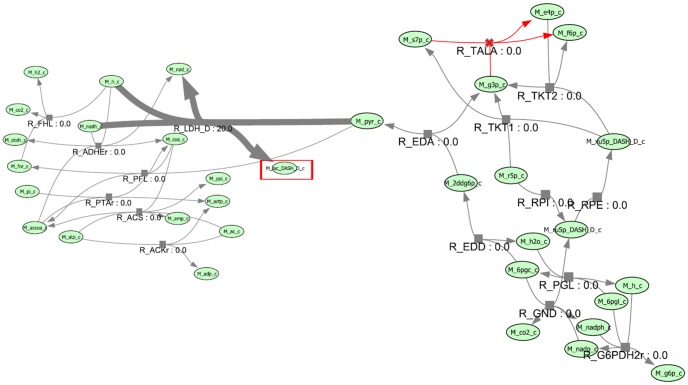
Flux map of the production of lactic acid.

DBFBA is then applied to identify the knockout strategy for producing lactic acid. Table 4 shows the growth rate of this method, which is 0.86719 and 16.1905 for BPCY. Figure 9 shows the comparison between different methods for growth rate and BPCY of lactic acid. The deletion of transaldolase (TALA) decreased the efficiency of gluconeogenesis which resulted in the increased concentration of phosphoenolpyruvate. Phosphoenolpyruvate is then converted into pyruvate and continues to convert into lactic acid. Knocking out acetaldehyde dehydrogenase (ACALD) which catalyzes the conversion of acetaldehyde into acetic acid eliminated the competing product, acetic acid. In addition, the removal of malate dehydrogenase (MDH) affects the oxaloacetate concentration which resulted in the increased concentration of pyruvate. As a consequence, the yield of lactic acid is improved. Figure 10 shows the flux map for the production of lactic acid. The flux map shows the flux increases to lactic acid.

### Production of Ethanol by *B. subtilis*

Table 5 shows the results of DBFBA and previous works to enhance the production of ethanol in *B. subtilis*. Ethanol is a volatile, flammable, colorless liquid, and it is a promising biofuel. Ethanol is currently used as an alternative fuel for gasoline worldwide. Therefore, ethanol was found to be the most suitable case study for this research.

**Table 5:** Comparison between different methods for growth rate and BPCY of ethanol by *B.subtilis.*

Method	Growth Rate (1/hr)	BPCY	List of knockout genes
DBFBA	**122.9657**	**1.1573e+05**	ALAD_L*, GK, sn-Glycero-3-phosphoethanolamine glycerophosphohydrolase
BAFBA[9]	122.8861	1.1154e+05	ALAD_L*, LDH_L, XYLI1, inosose 2,3-dehydratase

Note: The bold numbers represent the best result.

*Common genes for all methods. BPCY is in gram (gram-glucose.hour)^−1^.

**Figure 11:**
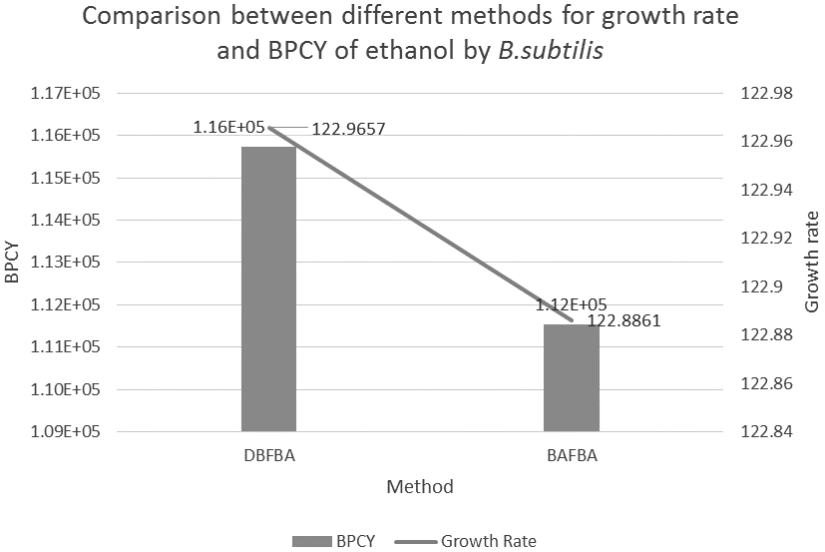
Comparison between different methods for growth rate and BPCY of Ethanol by *B.subtilis*. Note: BPCY is in gram (gram-glucose.hour)^−1^. Unit for growth rate is hr^−1^.

DBFBA obtained a better growth rate and BPCY which are 122.9657 and 1.1573e+05 respectively. Glycerol kinase (GK) and sn-Glycero-3-phosphoethanolamine glycerophosphohydrolase participate in glycerophospholipid metabolism, which involves the formation of glycerol. As stated by Pagliardini *et al.* (2013), glycerol is one of the main by-products in ethanol fermentation hence a substantial reduction of glycerol yield may lead to a significant increase in ethanol yield. In Pagliardini *et al.* (2013) experiment, a drastic reduction of the glycerol yield improved the yield of ethanol [19]. In addition, Figure 11 shows the comparison between different methods for growth rate and BPCY of ethanol, DBFBA obtains better results in both growth rate and BPCY compared to previous methods.

### Production of Ethanol by *C.thermocellum*

**Figure 12:**
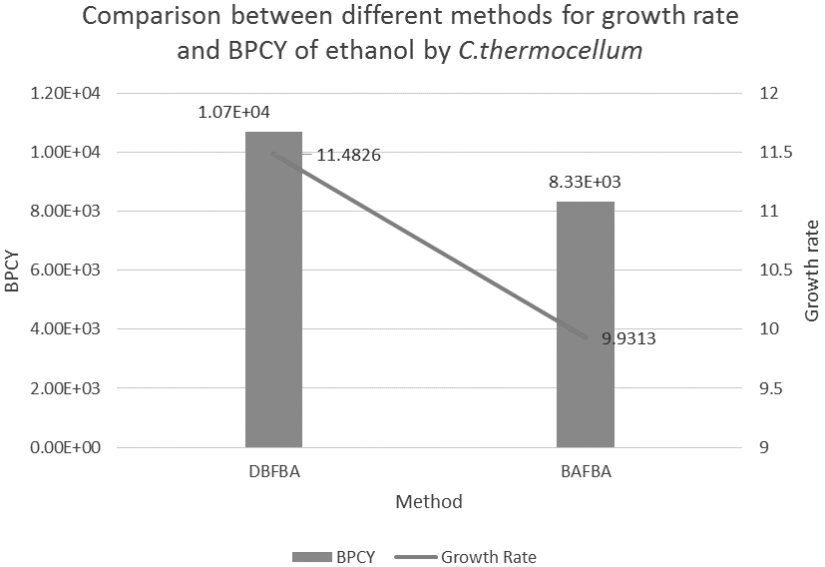
Comparison between different methods for production of Ethanol by *C. thermocellum.* Note: BPCY is in gram (gram-glucose.hour)^−1^. Unit for growth rate is hr^−1^.

**Figure 13:**
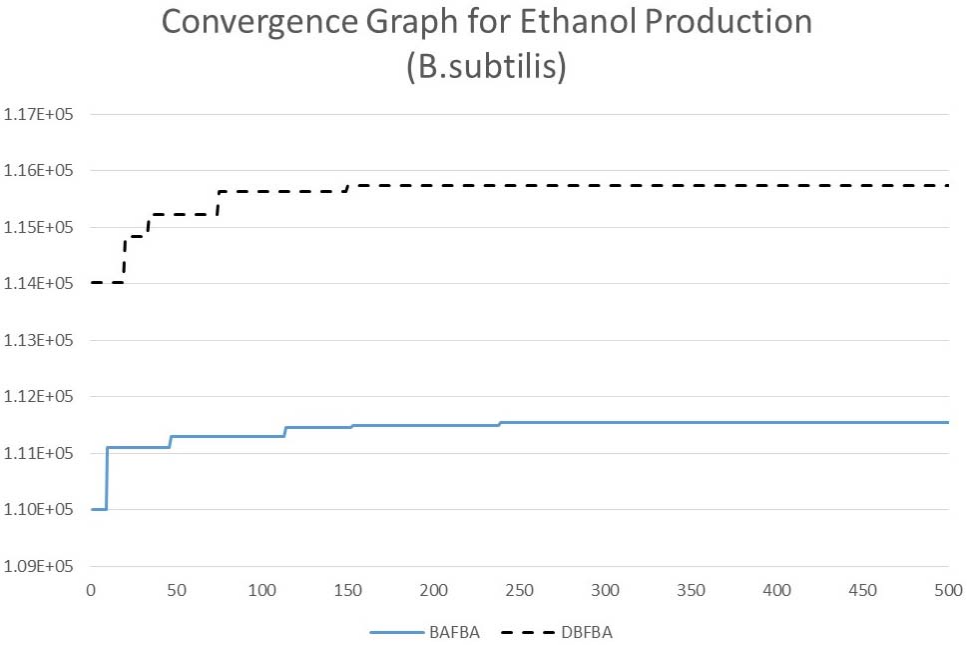
Convergence graph of different methods for ethanol production in *B.subtilis.*

**Figure 14:**
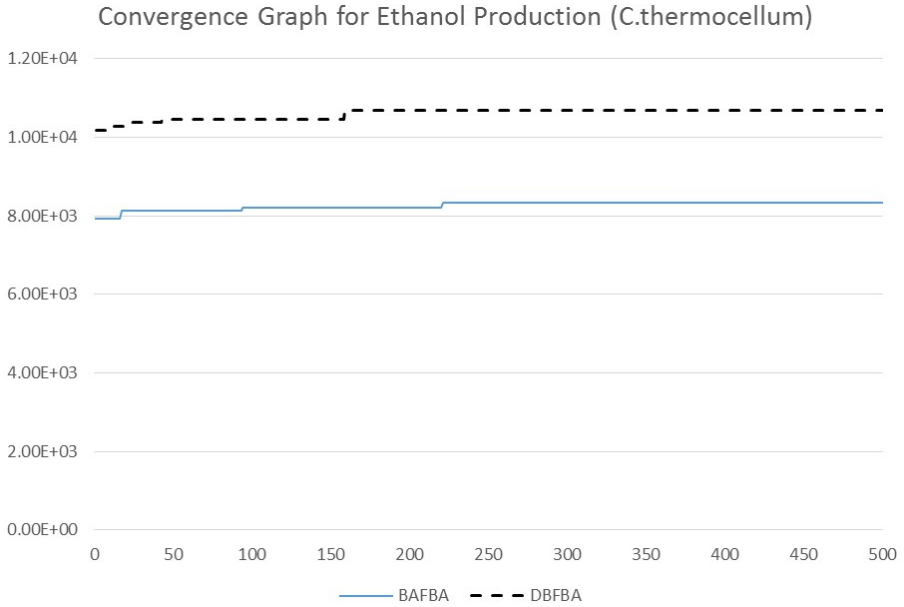
Convergence graph of different methods for ethanol production in *C.thermocellum.*

Table 6 shows the results of DBFBA and previous works to enhance the production of ethanol in *C.thermocellum*. DBFBA shows a better result for *C.thermocellum* model with the growth rate of 11.4826 and the BPCY 1.07e+004. Figure 12 shows the comparison between different methods for the growth rate and BPCY of ethanol. DBFBA shows that it could reach a higher value in both growth rate and BPCY than previous methods in the figure. The list of knockout genes shows the deletion of glycerate kinase (GLYCK), L-lactatedehydrogenase (LDH_L), and 2-Oxobutanoateformatelyase (OBTFL). Glycerate kinase participates in glycerolipid metabolism, which involves the formation of glycerol. In the experiment of Pagliardini *et al.* (2013), a drastic reduction of the glycerol yield improved the yield of ethanol due to which glycerol becomes one of the main by-products in ethanol fermentation [19]. As stated in Kim *et al.* (2012), lactate dehydrogenase (LDH_L) plays a key role in the fermentative metabolism in the metabolic engineering for ethanol production. The deletion of LDH_L inhibited the conversion from pyruvate to lactate, therefore more pyruvate was decarboxylated to acetaldehyde and further converted to ethanol [20]. 2-Oxobutanoateformatelyase is a key reaction for propionate metabolism. However, there is no specific report on the effect of 2-Oxobutanoateformatelyase for ethanol production currently. Figure 13 and Figure 14 show the convergence speed of DBFBA on ethanol production for *B.subtilis* and *C.thermocellum*.

**Table 6:** Comparison between different methods for growth rate and BPCY of ethanol by *C.thermocellum.*

Method	Growth Rate (1/hr)	BPCY	List of knockout genes
DBFBA	**11.4826**	**1.07e+04**	GLYCK, LDH_L, OBTFL
BAFBA [9]	9.9313	8.329e+003	MDH, G3PD1, PTAr

Note: The bold numbers represent the best result.

## Discussion

In addition, Table 7 shows the computational time comparison between DBFBA and BAFBA for 1000 iterations. The average computational time of DBFBA improved by 80%, 81%, and 87% compared to BAFBA result for 1000 iterations, respectively.

**Table 7:** Comparison between average computational time of DBFBA and BAFBA for 1000 iterations.

Model	Method	Computation Time (seconds)
*E.coli*	DBFBA	**2076**
	BAFBA [11]	10253
	OptKnock [5]	N/A
	SA + FBA [7]	N/A
*B.subtilis*	DBFBA	**4173**
	BAFBA [11]	22515
	OptKnock [5]	N/A
	SA + FBA [7]	N/A
*C. thermocellum*	DBFBA	**1350**
	BAFBA [11]	10282
	OptKnock [5]	N/A
	SA + FBA [7]	N/A

Note: The bold numbers represent the best result. N/A represents that the results are not reported in literature.

The results showed that both DBFBA and DB performed better than other algorithms. It can be concluded that the capability of DE algorithm in finding local optimum improved the performance of the original BA. The original BA had the problem of repetitive iterations of the algorithm in local search, where each bee keeps searching until the best possible answer was obtained. The proposed DBFBA solved the problem by implementing DE algorithm into the local search part. DE algorithm operates by maintaining a population of candidate solutions and creating new candidate solutions through the mutation and crossover operation of DE, and keeping the fitness candidate solution. Furthermore, DE algorithm is a heuristic approach mainly having three advantages; finding the true global minimum regardless of the initial parameter values, fast convergence, and using few control parameters. In addition, the OptKnock validation, which is integrated into BAFBA in this work, improves the reliability of BAFBA as the identified lists of potential genes have proven crucial in improving the desired phenotypes through literature [5], [19]–[20].

## Conclusion and Future Works

The development of accurate and efficient modeling methods as well as optimization methods in the field of metabolic engineering is crucial, as they will contribute significantly to the field of biotechnology. Consequently, it will lead to substantial economic gains in the production of pharmaceuticals, fuels and food ingredients. In this work, DBFBA has been proposed to be able to predict optimal sets of gene knockout strategies to maximize the production of desired phenotype and in the meantime, sustains the cellular growth. DBFBA improves the performance of BAFBA by implementing DE algorithm, which is indeed a promising algorithm in finding local optimum. Experimental results on *E. coli* model, *B. subtilis* model, and *C.thermocellum* model showed a hybrid approach, where DBFBA works as an effective tool in generating optimal solutions for the gene knockout identification, and therefore could be a useful tool in the field of metabolic engineering. The ability of DBFBA in predicting a set of genes that might affect the production of desired phenotypes is helpful to the biologists. Instead of testing single gene knockout effect on the production, the biologists can test on the effect of multiple genes knockout. Lastly, since user-friendly and publicly accessible web-servers represent the future direction for developing practically more useful models, simulated methods, or predictors [21], we shall make efforts in our future work to provide a web-server for the method presented in this paper.
